# Internal Cracks and Non-Metallic Inclusions as Root Causes of Casting Failure in Sugar Mill Roller Shafts

**DOI:** 10.3390/ma12152474

**Published:** 2019-08-03

**Authors:** Muhammad Jamil, Aqib Mashood Khan, Hussien Hegab, Shoaib Sarfraz, Neeraj Sharma, Mozammel Mia, Munish Kumar Gupta, GuLong Zhao, H. Moustabchir, Catalin I. Pruncu

**Affiliations:** 1 Department of Industrial Engineering, University of Engineering and Technology, Taxila, Pakistan; 2 College of Mechanical and Electrical Engineering, Nanjing University of Aeronautics and Astronautics, Nanjing 210016, China; 3 Mechanical Design and Production Engineering Department, Cairo University, Giza 12163, Egypt; 4 Manufacturing Department, School of Aerospace, Transport and Manufacturing, Cranfield University, Cranfield, Bedfordshire, MK43 0AL, UK; 5 Department of Mechanical Engineering, Maharishi Markandeshwar (Deemed to be University), Mullana 133207, India; 6 Mechanical and Production Engineering, Ahsanullah University of Science and Technology, Dhaka 1208, Bangladesh; 7 Key Laboratory of High Efficiency and Clean Mechanical Manufacture, Ministry of Education, School of Mechanical Engineering, Shandong University, Jingshi Road, Jinan 250061, China; 8 Laboratory of Systems Engineering and Applications (LISA), National School of Applied Sciences of Fez, Fez, Morocco; 9 Mechanical Engineering, Imperial College London, Exhibition Rd., London SW7 2AZ, UK; 10 Mechanical Engineering, School of Engineering, University of Birmingham, Birmingham B15 2TT, UK

**Keywords:** root cause analysis, non-destructive testing, destructive testing, optical inspection, microscopy, rejection rate, heavy manufacturing industry

## Abstract

The sugar mill roller shaft is one of the critical parts of the sugar industry. It requires careful manufacturing and testing in order to meet the stringent specification when it is used for applications under continuous fatigue and wear environments. For heavy industry, the manufacturing of such heavy parts (>600 mm diameter) is a challenge, owing to ease of occurrence of surface/subsurface cracks and inclusions that lead to the rejection of the final product. Therefore, the identification and continuous reduction of defects are inevitable tasks. If the defect activity is controlled, this offers the possibility to extend the component (sugar mill roller) life cycle and resistance to failure. The current study aims to explore the benefits of using ultrasonic testing (UT) to avoid the rejection of the shaft in heavy industry. This study performed a rigorous evaluation of defects through destructive and nondestructive quality checks in order to detect the causes and effects of rejection. The results gathered in this study depict macro-surface cracks and sub-surface microcracks. The results also found alumina and oxide type non-metallic inclusions, which led to surface/subsurface cracks and ultimately the rejection of the mill roller shaft. A root cause analysis (RCA) approach highlighted the refractory lining, the hot-top of the furnace and the ladle as significant causes of inclusions. The low-quality flux and refractory lining material of the furnace and the hot-top, which were possible causes of rejection, were replaced by standard materials with better quality, applied by their standardized procedure, to prevent this problem in future production. The feedback statistics, evaluated over more than one year, indicated that the rejection rate was reduced for defective production by up to 7.6%.

## 1. Introduction

Three-roller sugar milling is a global practice to extract the juice by crushing the sugar cane. In this process, the mill roller shaft is a critical component of the sugar mill, and its failure leads to downtime for the whole sugar industry. For this reason, the sugar roller mill shaft is considered a major cause of concern. The sugar mill roller is made of steel alloy material shafts, manufactured by shrinking the cast iron ingot in the foundry shop [1]. The manufacturing of the sugar mill roller shaft is costly due to its immense weight and size, being fabricated from several tons of raw material and factory resources [2]**.** The total weight of a casted manufactured ingot varies in the range of 6~9 tons. The initial ingot size made of cast iron has a length around 2700~3400 mm and a diameter around 600~650 mm. However, the final finish-machined shaft (mill-drive shaft and shell) weight varies in the range of 2.5~4 tons (where the maximum dimension of the mill drive shaft reaches a length of 2200 mm and a diameter of 500 mm [3]. A significant reduction of material from the finished shaft is associated with the wastage of material in key manufacturing processes, such as the casting gate system, pipes, fettling, and rough and finish machining. This explains the high cost of the manufactured shaft. The massive size of an ingot makes a fast evaluation of ingot quality difficult. It is well known that the casting for one sugar mill roller shaft takes almost 2~2.5 weeks to lower down its temperature, equal to forging temperature, until the initial quality check is carried out after rough machining of the forged shaft. Further, the average life cycle of a sugar mill roller shaft is approximately one year. After this period, surface and sub-surface wear and tear starts to occur due to abrasive wear, continuous fatigue, or internal crack propagation in the shaft. Such type of failures affects the system’s reliability and sometimes minimizes the service life by up to half [4]. The service conditions of roller shafts are harsh, abrasive, and compressive, owing to the relative motion of paired massive shafts under a humid and corrosive environment. The degradation of shaft material can be associated with excessive fatigue, internal cracks, nonmetallic inclusion, corrosion, and wear. It was noted that at the end of every season, the shaft reduces its diameter up to 20 mm due to corrosion and abrasive wear produced in each season. Further, from its finished shaft diameter of around 500 mm, it reduces gradually to 480 mm, 460 mm and 440 mm, respectively, which reduces the weight of the final shaft [5]. Usually, three maintenance sessions are conducted until external abrasive wear reaches 1.8% depth from the original diameter of the shaft. The restoration/maintenance of the shaft includes machining of worn surfaces and grooving new surfaces with a better surface finish. The restoration and extra machining explore new sub-surfaces, thereby reducing the diameter, which is the ultimate loss of productivity. From an economic perspective, this restoration session can be performed three times until the diameter of the shaft approaches lower than 300 mm, and the mill roller shaft is discarded for future use [5]. Therefore, composition, internal cracks, and undesirable inclusions are the key factors that influence the life cycle of the sugar mill roller shaft.

**Figure 1:**
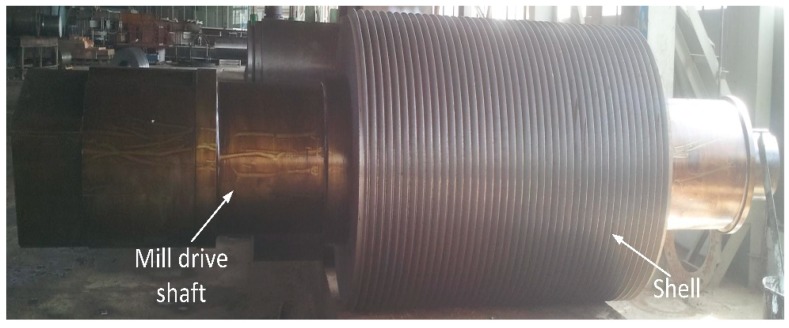
Sugar mill roller shaft with final groove ready for shipment.

The favourable specifications for the mill driveshaft are open grains of cast iron from an initial ingot, made by rough machining [6]. The material should be free of internal blowholes, cracks (either surface or sub-surface), non-metallic inclusions and micro-grooves on the surface, in order to reduce the abrasive wear. Due to high load, complex stresses and rough finishing, higher tensile strength materials are usually preferred [7]. It is pertinent to mention that abrasive wear can be reduced by making a groove on the finished shaft with an acceptable surface roughness of less than 0.8 μm (Figure 1)**.** The optimum groove angle for a sugar mill roller shaft varies between 45 and 55 [3,8]. Fine grooves can extract the sugar in a better way. Three types of grooves are commonly manufactured into finished sugar mill roller shafts;

- Fine grooves, containing 5–20 mm of pitch
- Medium grooves, containing 20–50 mm of pitch
- Coarse grooves, containing >50 mm of pitch

The removal of defects by reworking in production industries can add an extra cost that is transposed in the final product cost [9]. When demand increases and bottlenecking is at the peak, defects will result in loss of revenues. If defects are not adequately inspected, the defective product results in an unsatisfied customer, which is the worst outcome of the defective product [10]. The main reasons behind the defective product are the use of raw materials with poor quality, lack of latest equipment, training, and regular maintenance of the available equipment [11]. Economical manufacturing can be ensured by reducing the unit production cost by lessening the rework, initial product cost or cost due to rejection of the final product. The production quality in terms of meeting the customer needs and specifications is also one of the critical aspects [12]. A rejection can be defined as an imperfection or deviation of a product or component from its design specifications. A product is a scrap if it is a part that does not confirm the required specifications**.** The cost, time, and product quality are the critical aspects of any manufacturing industry. The on-time delivery of products can be ensured by reducing the rejection time. For on-time delivery of sugar mill roller shafts, defect-free production is necessary.

### 1.1. Internal Cracks and Porosity

For quality checks, grain structure, internal cracks, and chemical composition could play a crucial role in the acceptance/rejection of the final sugar mill roller shaft. Therefore, the initial free defect casting is necessary for on-time delivery of the products. A recent study endorsed the most desired material for heavy industries (42CrMo4), which can be used to manufacture sugar mill drive shafts. The abovementioned study dedicated much attention to this material’s specifications under various quality tests, owing to the high demand for quality from sugar industries to manufacturers while purchasing the shaft. It was identified that quality check results are mostly requested from the sugar industry, while a precise specification of the final product is hardly requested [13]. Several destructive/non-destructive techniques are reported in literature, focusing on the major causes that lead to defective production, such as magnetic particle testing [14], ultrasonic testing [15], crystallographic testing [16] and scanning electron microscopy (SEM). The in-depth porosity effect on the fatigue life of the A319 alloy was discussed by Zhang et al. [17]. Findings showed a reduction in fatigue life at a high porosity level. This can be associated with the large-scale distribution of porosity, which allows the initiation of cracks, leading to the failure of the product.

Fuller et al. [18] analysed a defective shaft of stainless-steel (AISI-304) material. During the research process, a conventional 14-step root cause analysis (RCA) technique was applied. These steps consist of observation, information collection, visual observation, record keeping, non-destructive testing, mechanical testing, selection/preservation of fracture surfaces, macroscopic/ microscopic testing, metallography, failure mechanism determination, chemical analysis, mechanical analysis, simulated service conditions and final analysis and life cycle reports. This failure analysis is described as a complete set of standardized quality procedures. It can highlight the key reasons for failure and provide a method to avoid recurrence in future.

Further, a failure and root cause analysis of a vehicle drive shaft was performed by Zhao et al. [19]. They investigated the failure mode of the shaft in order to detect the root cause of failure. Specifically, they applied macroscopic and microscopic analyses to the morphologies of the fracture surface, chemical composition analysis, metallographic testing, mechanical properties analysis, and finite element analysis (FEA) on the rejected drive shaft. Their research outcomes presented the fatigue as the dominant mechanism of driveshaft failure. The primary cause of fatigue cracks was linked to high-stress concentration generated at the root fillet due to small geometrical dimensions.

Moreover, high manufacturing tolerances can promote high-stress concentration and thus accelerated crack initiation and propagation. Using the same approach, Ni et al. [20] performed a failure analysis of a boric acid recycle pump plant. The root cause analysis included material, metallographic and mechanical property testing; wear observation; micro-zone analysis and mechanism analysis. During the analysis, the primary causes of failure were identified as triple-stress concentration, surface defects, and the aggregation of inclusions [21].

The use of steel grades in mill drive shafts is a frequent practice in the industry. The analysis of designed parameters for such products is fundamentally essential. The time for pouring, moulding and shaping plays an essential role to enhance the performance of the casting process. The present research proposes a typical geometry that is finished by mould operation. In order to reduce the occurrence of potential cracks during manufacturing, an optimized design for a mill drive shaft is selected. All the parameters used here were identified and prioritized for improvement. Numerous researchers have performed failure analysis of products developed by manufacturing. Rokhlin et al. [15] accomplished an analysis of compact roll failure for rolls that fractured in service after a short period. The internal bearing of the shaft experienced wear after a long service time. During the process of depositing a thin film of material on the substrate, a crack was produced near the shaft curvature radius. This failure analysis was carried out by performing a liquid penetrant test, fracture analysis, and metallographic tests. Shafts are used for transmitting torque under different operating conditions [22]. These shafts are exposed to various kinds of stresses (such as tension, torsion, and bending) due to axial or radial loads. When mechanical and metallurgical stresses are combined, the probability of crack initiation and propagation leads to the failure of the component [23].

There are numerous studies to analyse the failure mechanisms of shafts. Out of all these studies, one was completed by Prasanthi et al. [24], who observed the sodium leak. There, the reducer promoted a reduction in the carbon meter circuit of a dynamic sodium loop used for high-temperature sodium trials. A smaller amount of carbon meter was activated at a temperature of 623 K, after 6600 h of testing. The accurate metallurgical classification was carried out to apprehend the cause of failure. Spectrometry results revealed that the reducer was made of 304 stainless steel, containing 0.08 wt.% of carbon. Cracks were identified at 4 mm on the weld interface, where the internal structure was fully exposed. Electron microscope inspection confirmed the presence of (Fe, Cr) 23C6 carbides in the grain boundary. Therefore, the rejection was linked to low-temperature sensitization, followed by intergranular stress corrosion cracking.

### 1.2. Non-Metallic Inclusions

Non-metallic inclusions can be defined as undesirable metallic or non-metallic chemical compounds, such as oxides, nitrides or sulphides, that contain an extra compound such as carbonitrides, oxy-sulphides and others. Based on the origin of foreign particles, they can be categorized into endogenous inclusions and exogenous inclusions [25,26];

- Endogenous-type inclusions form when high-temperature liquid metal reacts with some deoxidizers, such as aluminum or silicon, during the process of desulfurization. The chemical reaction makes a new product, which cannot be dissolved through slag. However, their fractions can be minimized through the vacuum-arc melting of the secondary filtration of melt-metal.
- Exogenous-type inclusions transfer into the melted metal from external sources, such as flux material, initial raw material, refractory material, sand or slag covering the metal. The final size of the exogenous inclusions is bigger than that of endogenous inclusions.

Non-metallic inclusions favour crack-propagating sites, especially in high-strength steels and Nickel-based alloys. Non-metallic inclusions are the leading cause of failure reported in the literature regarding crankshaft, gears, roller shafts, and materials with high fatigue limit [27,28]. The crack initiates from the site under high local strain, which can increase the probability of subsurface cracks/defects. It can be associated with a mismatch of composition between foreign particles and the metal matrix. Non-metallic inclusions produce mismatches such as stiffness mismatch, thermal expansion mismatch, ductility mismatch, and chemical mismatch. For example, the stiffness of a material is characterized by Young’s Modulus (E) [29]. Therefore, ‘E’ can be under- or overmatched between melt metal and inclusion. In the case of Al_2_O_3_ inclusion, stiffness is overmatching, and MnS is under matching. It is difficult to match the E for uniform stresses generated on the metal matrix that show higher strain than the inclusion. They can develop a strain concentration zone that produces residual stress around this region [30] and initiates in-depth mini-cracks at the metal– inclusion joint interface. Hence, the initial small crack is already present before applying an external load.

Besides the nature of inclusions, the size, localization, and shape of particles also have a significant effect. There is a clear relationship between the size of inclusion and fatigue strength. Findings have shown that the bigger the size of inclusion, the lower the fatigue strength of the final product [31]. Similarly, a longer length of inclusion is associated with a longer crack length. The size and length of inclusion increases the possibility of crack propagation rate and crack driving force, but also decreases the life of the product. Besides, the morphology and localization of the inclusion concern the surface and spatial distribution, with direct effects on the load direction applied on the workpiece. Yong et al. [32] detected a crack initiation mechanism in low alloy steel. Findings concluded that cracks initiated due to sub-surface inclusions, in a ‘fish-eye’ trend. The size of the fish-eye crack pattern increases with the increasing depth of inclusions on the surface. Furthermore, the simulated results suggested that a large size of inclusion, small grain size, and high strength of molten metal may promote crack formation.

### 1.3. Research Objective

The current research work aims to elucidate the “root cause analysis for failures” of a mill drive shaft in a Heavy Mechanical Complex (HMC), Pakistan. The sugar mill drive shaft is considered as one of the critical products of the industry mentioned above. Before this research, the rejection rate of the mill drive shaft was nearly 14.3% per year. Therefore, it is a huge concern from an economic perspective for such an industry. This high rejection rate, along with labour, overhead, transportation, and manufacturing, can increase the product cost considerably. If the product is rejected at any stage, e.g., during forging or heat treatment, it increases the lead time as well. The principal objective of this research was to achieve a reduction in the rejection rate for the mill drive shaft by finding out the root causes of defects. In order to find out the causes of defects, destructive and non-destructive tests were performed. By finding out the origins of defects, the defect rate can be reduced or eliminated. The decrease in the defect rate will help to reduce the lead time, hence increasing the profitability of the organization.

## 2. Research Methodology

The primary function of the mill drive shaft is to crush the sugar cane and separate the juice. Sugar cane enters through three mill drive shafts and is crushed between the feed-top roller. The juice is collected through the trash plate, and bagasse comes out from the top-discharge roller. The bagasse is squeezed once again through a three-mill roller system. In this way, more than 96% of the sucrose is removed from the sugar cane. The power is provided on the top roller, which drives the other two rollers. The present research involves the manufacturing of a mill driveshaft made of 42CrMo4 from 6~9 tons of raw material (scrap 98.3% and crude iron 1.7%) in the shredded form [5,33]. The casting was done through an electric arc furnace (EAF). The raw and flux material for casting includes low manganese pig iron, ferroalloys, scrap steel, carburizing, ferrosilicon, ferromanganese, calcium carbonate, lime, fluorspar, and so on. The chemical composition of the required standard was achieved at a temperature of 1500 °C. After that, ferroalloys with a higher melting point, such as FeMo and FeW, were added into the charge at 1565 °C. In order to remove phosphorus, hydrogen, and nitrogen, and for the purpose of refining, oxygen was blown into the steel with the pressure at 0.8–1 MPa, and to ensure the decarburization rate, at least 0.60% carbon was inserted per hour. The meltdown slag was continuously removed. Later, lime was added for better fluidity, with high FeO content (around 20%) to ensure proper dephosphorization. The deoxidizing period was commonly 40–60 min. Deoxidizing ferroalloys were added, such as Silicomanganese (SiMn) at 3kg/ton, Aluminum (Al) at 0.8kg/ton, Ferrosilicon (FeSi) at 3kg/ton, and calcium silicon (CaSi) at 1kg/ton, only if Sulphur exceeded its limit and ferro-manganese (FeMn) exceeded 1% of raw material weight. There were various temperature and pouring standards. For example, 42CrMo4 materials’ melt preparation time was 8hr, the furnace temperature was 1670~1680 °C, and ladle temperature was 1590~1610 °C. The melt was poured in master molds at a pre-pouring temperature of 80 °C, and the pouring time was 3 ton/minute. This resulted in the attainment of a cylindrical/hexagonal-shaped bar of 1000 mm diameter and 1200 mm length of the ingot. After the pouring into the matter molds, the total time to the hot top level was 6 minutes, and steel surface inside the hot top had to be covered with a sufficient amount of “Ferrux” insulating powder, i.e., 2 kg per ton of ingot, and later with fire clay. The casting was allowed to cool at room temperature until it solidified and attained forging temperature. After solidification, the ingot was removed from the mold body. As the temperature of the ingot reached 850 °C, it was heated in a furnace for 4 hours to attain the 1150°C forging temperature. It is pertinent to mention that the initial ingot temperature of less than 600 °C is considered as a cold-ingot. The forging was carried out through a 1000-ton hydraulic press to improve the grain structure and to make a cylindrical shape of mill drive with a diameter of 700 mm. Open die forging was conducted for the ingot, through flat dies with no complex profile. After forging, anti-hydrogen heat treatment was carried out for 11 hours. This was performed at different temperatures varying from 800~650 °C to improve the grain structure, mechanical properties, and stresses. The primary purpose of anti-hydrogen heat treatment is to eliminate hydrogen and nitrogen as much as possible. The standard composition and mechanical properties achieved are summarized in Table 1.

**Figure 2:**
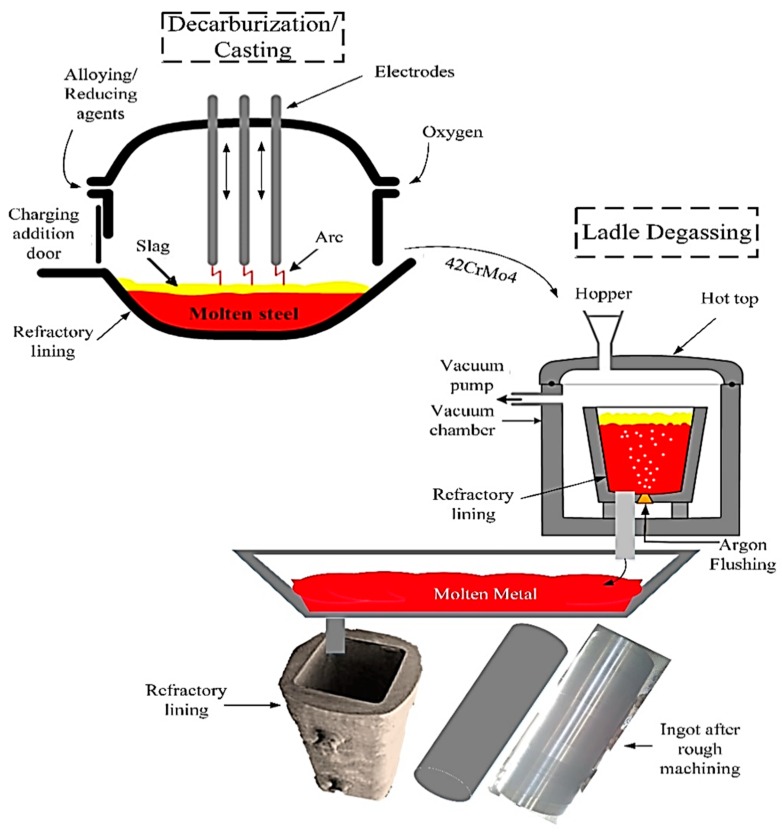
Possible sources contributing to the rejection of the roller shaft.

Figure 2 highlights the procedure to make the roller shaft ingot. Throughout the process, factors involved in the casting process are highlighted that can contribute to the quality as well as the rejection of the sugar mill shaft. The major sub-processes in casting are de-carburization/casting, ladle degassing, the refractory piping system, the ingot mold body, and the rough-machined ingot ready for the initial quality check.

**Figure 3:**
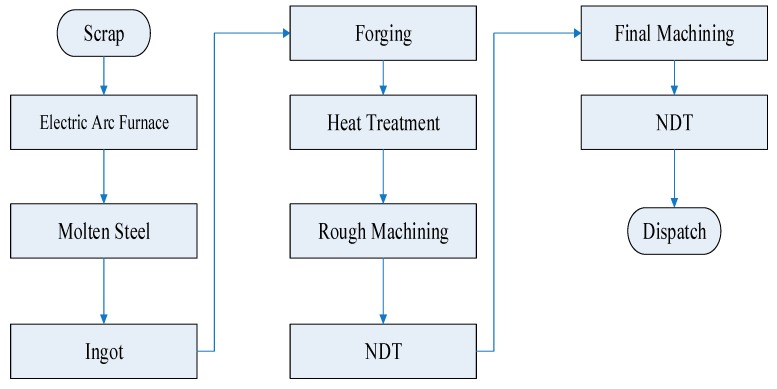
Flow chart depicting critical processes in the manufacturing of the sugar mill roller shaft.

Further, rough machining was performed to remove the unwanted material as the ingot reached room temperature. Rough machining was used to attain a smooth surface to apply further quality tests to ensure that any surface/subsurface cracks and non-metallic inclusions were <~300μm. Figure 3 presents the scheme of the manufacturing process on the mill drive shaft.

### 2.1. Non-Destructive Testing

Non-destructive testing is a procedure of testing/inspection or evaluation under the observed components or assemblies to detect any discontinuities, flaws, or cracks on the surface and subsurface, without destroying the serviceability of the component/assembly. Test methods are named based on the test equipment or penetrant medium used to perform the test and to detect defects such as liquid penetrant testing (PT), ultrasonic testing (UT), electromagnetic testing (ET) and so on. In this investigation, the following non-destructive tests are considered to find the desired objectives.

#### 2.1.1. Liquid Penetrant Testing (PT)

**Figure 4:**
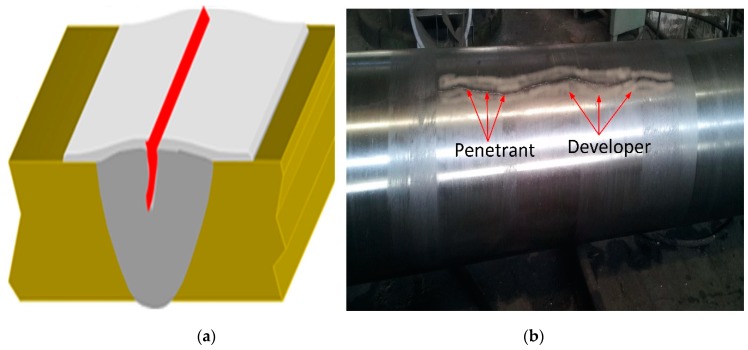
(**a**) Schematic diagram depicting the application of cleaner, penetrant, and developer; (**b**) Sugar mill roller shaft after penetrant testing (PT)**.**

Liquid penetrant testing is the most popular non-destructive technique in the industry, owing to the fact that it is cheap, versatile, and applicable at any stage of the manufacturing process. The main feature of penetrant testing is to detect surface breaks/flaws by flowing a thin liquid into the flaw and drawing the penetrant out by means of a developer [34]. Stainless steel, steel, aluminum, rubber, and cast iron are commonly inspected after casting or forging with this technique [35]. In this test, a dust cleaner (SKC-1; Magnaflux; ITW India Private Limited) was used to remove dust particles, oil, or grease particles. A penetrant (SKL-SP1; Magnaflux ITW India Private limited) was sprayed from aerosol, a dwell (soaking) time of 12 minutes was allowed for it to permeate well into the crack/flaw. After the dwell time, the penetrant was removed with a clean rag. After that, the thin coating of the developer (SKD-S2; Magnaflux ITW India Private limited) was sprayed, with a dwell time of 25 minutes for it to exit the cracks and to create an indication line on the developer (Figure 4). The part was examined critically to find the penetrant indication line growing with time as penetrant bleeds out of cracks. The accessible surface was inspected as per ASTM-E165 standard.

#### 2.1.2. Ultrasonic Testing (UT)

**Figure 5:**
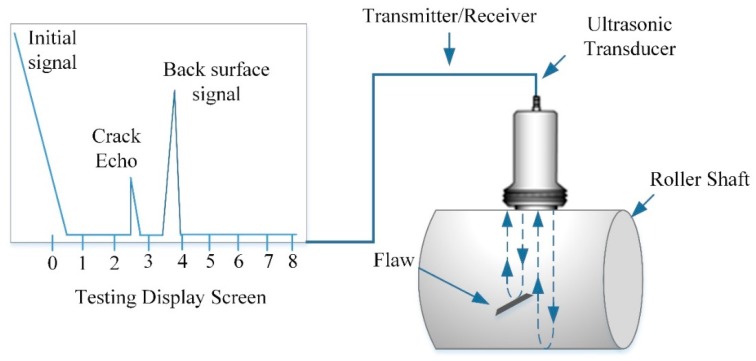
Schematic diagram of the ultrasonic testing (UT) set-up, depicting the test screen and compression probe.

Ultrasonic testing belongs to a non-destructive inspection method that permits a high-frequency sound wave to be passed through a workpiece material under inspection, and the reflected sound is detected. It permits the evaluation of crack activity as a function of ultrasonic wave propagation under different loading conditions. Most of the time, ultrasonic testing is carried out at a frequency of 0.4~20 MHz, which is beyond the human hearing range (20 Hz~20 kHz). Mechanical vibrations of medium particles are named ultrasonic waves, having a particular frequency, velocity and amplitude. Here, the amplitude is a particle’s displacement from a mean position; frequency is cycles per seconds; and wavelength is the length of a cycle. The relationship between velocity, frequency and wavelength is given by v = fλ. The UT inspection system incorporates a portable probe of transmitter/receiver, transducer, and a display screen (see details in Figure 5). A pulse is generated by imposing an alternating voltage that activates a piezoelectric probe crystal to produce signals by coupling the probe to it. The sound waves emitted from the boundary of the workpiece reveal any crack/cracks that is received as feedback from the probe. Through a reverse piezoelectric crystal effect, received waves are converted into voltages and displayed on the screen. Here, an ultrasonic system was used (HY-350; Suzhou De Sisen Electronics Co. Ltd, Jiangsu, China) that permits a detection range of 0~6000 mm thickness, and a dynamic range of >32 dB, with a single probe. The sensitivity of the system is 0~110dB in order to overcome the effect caused by the surface roughness of the detected workpiece. The transducer system (HY-350; Suzhou DeSisen Electronics Co. Ltd, Jiangsu, China) production is driven by a pulser with a 2.0-MHz frequency-based ultrasonic energy. The system resolution is >40 dB. The location of the crack is obtained by moving the probe. The crack waves are produced from zero to maximum, as the probe moves from the defect-free region to the defective region. The characteristics of the crack wave obtain the location of cracks.

The distant amplitude correction (DAC) curve is a technique to compensate for the pulse-echo response linked to reflector decreases, which are due to the increase in the distance of the reflector from the probe. The transmitted beam travels from the probe to the reflector and reflects on the probe by dropping sound energy that hits the reflector when received by the probe. DAC is plotted from peak amplitudes of reflectors possessing a similar area and at different distances, by following the ASTM E1158. DAC permits, though the reflected signals, the evaluation of similar discontinuities, where signals’ attenuation concerning depth is related. DAC is plotted based on the following criterion.

The DAC is plotted with the step-type flat bottom hole (FBH) calibrated reflector. The dimensions of the used step-type flat bottom hole (FBH) reflector are given in Table 2 below.

The DAC is drawn with the above-mentioned step-type FBH reflector, by keeping the back-wall echo at 80% of the full-scale height from the nearest/smallest FBH (i.e., reference FBH at 125 mm). After that, the back-wall echo from the other two reflectors (i.e., FBH at 250, and 375 mm reflectors) is measured, keeping the same gain setting of amplitudes and DAC drawn on the display screen. DAC is plotted from the peak amplitudes of reflectors possessing a similar area and at different distances. The acceptance criteria for the part are given below:

- No indication should be above the reference DAC curve.
- Two or more indications should not exceed 50% of the DAC curve within 12.7mm of each other, in any direction.

After plotting the DAC curve, the scanning of the component is carried out, keeping the same settings of the equipment. In order to ensure 100% area coverage of the component, scanning is carried out with 10% overlapping of the probe area.

### 2.2. Destructive Testing

Destructive testing involves the breakage of component/product into specific samples to determine in-depth structural properties, strength, hardness, and toughness of the material. Such a type of tests is commonly applied as a trial for mass-produced components/items, in which the destruction cost of specific items is economically feasible. Some destructive tests are named based on equipment and specific objectives, such as magnetic particle testing (MT) and metallographic tests, including microstructure and macrostructure analyses.

#### 2.2.1. X-Ray Spectroscopy Analysis

The quantitative spectroscopy technique is a chemical micro-analysis that allows the detection of the surface composition of the workpiece materials. In this technique, an electron beam bombards x-rays onto workpiece samples. The bombarded x-ray causes the ejection of an electron from the workpiece atoms. The energy of the emitted electron is a core function of its binding energy, and a specific characteristic of the element from which it is emitted. As the electron is ejected through an incident-ray, another electron comes to fill the hole. The x-ray energy detected is characteristic of the composition of elements from which it was emitted. The x-ray energy spectrum versus count is used to evaluate the composition of elements. In this research analysis, small samples were prepared from the portion of subsurface cracks detected under UT. Energy Dispersive (X-Ray) Spectroscopy (EDX-6000B; Sky ray Instrument, Suzhou, China) was used to detect the composition of the samples.

#### 2.2.2. Magnetic Particle (MT) Testing

**Figure 6:**
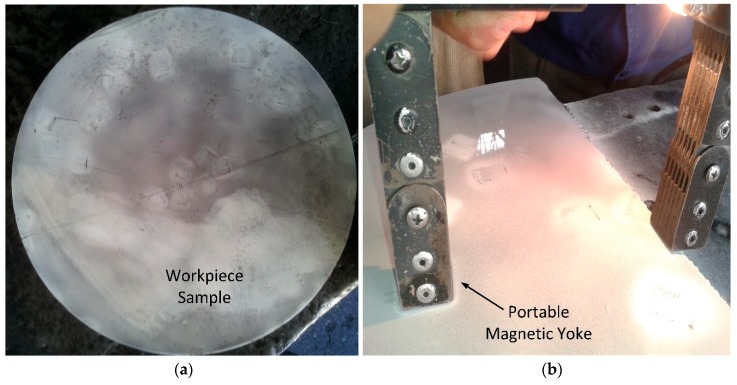
(**a**) Prepared sample for magnetic particle testing (MT); (**b**) MT yoke system to detect cracks.

Magnetic particle evaluation belongs to destructive testing and is commonly performed by generating a magnetic field for ferromagnetic-based raw, semi-finished or finished materials. MT permits the detection of surface and subsurface cracks and discontinuities, which reduce the fatigue strength or eventually lead to part failure as per ASTM-E709. In the MT visual inspection technique, a portable yoke-based magnetic testing set-up (CDX M-1; Testech; Beijing, China), which works with AC (alternating current), is used to find the flaws/cracks. The yoke system is an electric stand wrapping an electrical coil around a piece of soft ferromagnetic steel. Fine black iron particles are spread over the workpiece surface. When testing black particles, white paint is applied on the plate surface to attain better contrast to show cracks. Also, iron particles are sprayed with fluorescent and viewed under ultraviolet light to attain a glow of cracks. Black particles are sprayed with liquid suspension, enabling the free flow of particles over the workpiece surface. Modern solvent carriers are specifically designed with properties that have flash points above 93^o^C and keep nocuous vapors low. High magnetic permeability is essential because it makes the particles attract easily to the small magnetic leakage fields from discontinuities, such as flaws/cracks. Fine iron particles accumulate at the poles of a magnet; however, in any discontinuity/crack, iron particles accumulate at the flaw/crack location and adopted same orientation. Low retentivity is vital, because the particles themselves never become strongly magnetized so they do not stick to each other or to the surface of the part. Due to the large diameter, MT was performed in steps to detect all the surface of the workpiece. The MT testing application set-up is depicted in Figure 6.

#### 2.2.3. Metallographic Testing and Microstructure Analysis

Metallographic testing consisted of cutting the sample from the observed part, mounting it with resins, coarse grinding for the required specification, mounting and polishing with alumina powder or diamond paste, etching in dilute acid, washing with alcohol, and drying to observe the surface in a variety of tests. This procedure allows us to observe the microstructure under high magnification, (50×~1000×) through a microscope. Also, large magnifications can be attained through scanning electron microscopy (SEM). Microstructure analysis is commonly used to determine the morphological structure of a material. The microstructure analysis was performed using an optical microscope ARTCAM (130-MT-WOM; ART-RAT; Yokohama, Japan) to observe the macrostructure evolution of the investigated material. Furthermore, SEM (S-4800 II FESEM; Hitachi High-tech Corporation, Japan) was used to analyze and identify the nature of cracks and foreign particles in the surface/subsurface of the workpiece material. In order to observe the microstructure properties of the sample, specimens were ground with fine SiC waterproof papers (120~1500 grit size) to attain a flat surface and were lubricated with distilled water. After cleaning, specimens were polished with a separate wheel using 6μm of diamond paste and later 1μm of diamond paste (large to small size particles) to attain a mirror-like surface. After examination of the features, specimens were etched to develop additional contrast to observe the microstructure. The samples were hotly etched in diluted acid (2%Nital for steel) for about 10–15 seconds and scanning electron microscopy (SEM) was used for phase analysis. The samples with small or irregularly shapes were mounted with Bakelite resin in a hot mounting press. The analysis was performed in samples with nominal dia. of 20 mm length and 10 mm width, at different magnifications (100×, 200×, and 400×).

## 3. Results and Discussion

This section highlights the presentation of destructive and non-destructive test results. Moreover, the analysis of the results is discussed in detail.

### 3.1. Non-Destructive Testing

#### Ultrasonic Testing (UT)

**Figure 7:**
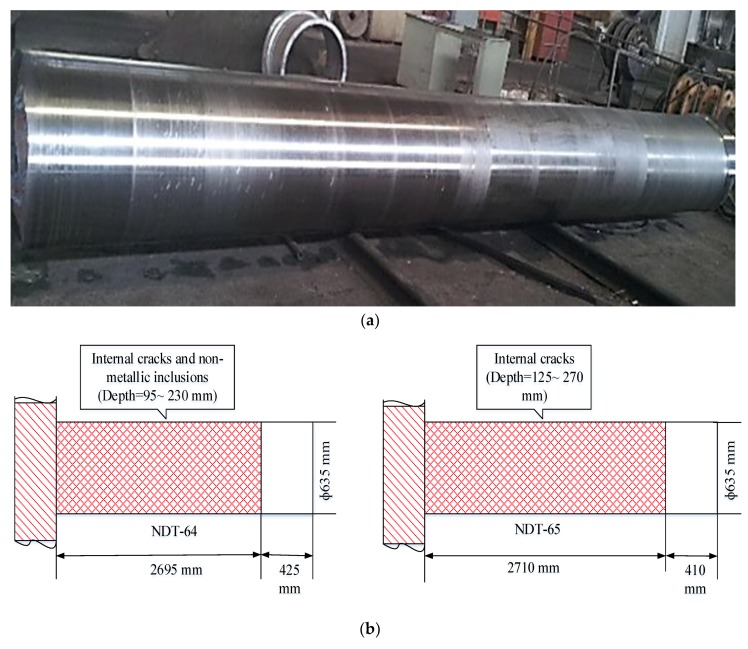
(**a**) A rough machined shaft that was rejected after UT inspection; (**b**) UT report locating internal cracks and foreign particle inclusions under duplicate measurements.

During the scanning of the component, a few indications were found to be above the DAC curve, within 12.7 mm. Based on the indication pattern, the scanned component on the DAC curve showed an indication of flaw. Ultrasonic testing precisely indicates subsurface cracks/discontinuities and the possibility of nonmetallic inclusions [36]. UT measurement was performed twice to validate the presence of cracks and inclusions. The presence of cracks was observed under the supervision of the local industry quality control department non-destructive testing (NDT-64). The NDT-64 reports of the shaft under observation showed non-metallic inclusions and deep internal cracks at a depth of 95~230 mm, randomly distributed throughout the shaft length of 2695 mm (details in Figure 7). For further clarification, UT testing was performed under the supervision of a multi-national quality control department (NDT-65). So, rejection was ensured with the identification of a flaw with its position. NDT-65 depicted internal cracks within the depth of 125~270 mm, randomly distributed through the length of 2710 mm. Based on the detection of cracks and foreign particles, the rough machined ingot was rejected.

### 3.2. Destructive Testing

The rejection decision requires further analysis through destructive testing to determine the root causes that led to the potential failure. Randomly collected data from last year showed that significant reasons behind the rejection of shafts were non-metallic inclusions (50%), piping (31.25%), and surface/subsurface (18.75%). The previous data clearly showed that non-metallic inclusions played a leading role in the rejection of shafts. However, it was essential to find substantial evidence associated with the cause of the rejection of this shaft. During the destructive testing, the rough machined shaft was sectioned from the middle of the shaft into two pieces. After that, the sample pieces of almost 635 mm diameter and 10 mm thickness were separated. They were further divided into small pieces to obtain the small size samples of the specific portion of the shaft within 100~200 mm depth (to attain a more specific region with higher cracks and inclusion), highlighted under ultrasonic testing.

#### 3.2.1. X-ray Spectroscopy Analysis

X-ray spectroscopy analysis gave the composition of material through samples from different locations of the shaft to ensure the composition of the shaft material 42CrMo4 provided in Table 3. Four samples from different locations were collected to ensure the chemical composition of the shaft under spectroscopic analysis. A comparison of spectrometry results with the standard composition (Table 1) authenticate that the chemical composition of all the components is within the range of the standard composition of 42CrMo4. It underscores that there is no issue with the composition of the shaft material.

#### 3.2.2. Magnetic Particle Testing (MT)

**Figure 8:**
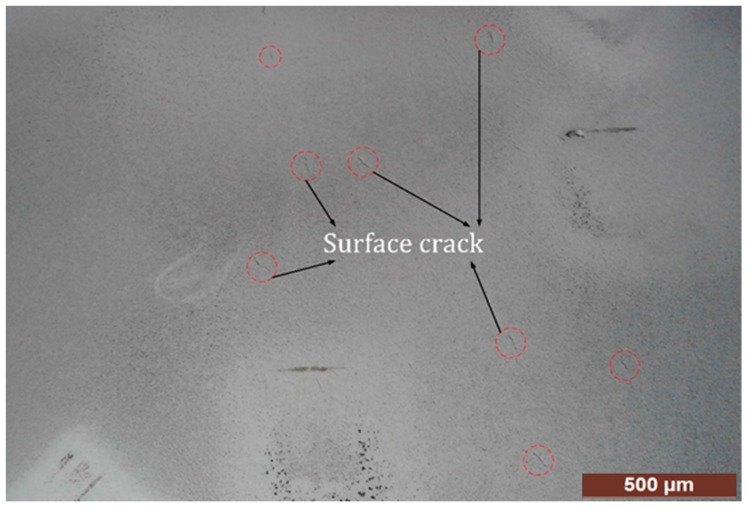
Surface cracks after performing magnetic particle testing.

After preparing small size samples for destructive testing, magnetic particle testing was applied to detect discontinuities and crack dispersion on the specific portion of the shaft highlighted under UT. In the MT procedure, any distinct variation or discontinuity that distorted the magnetic lines, named flux leakage, was observed due to surface/subsurface cracks identified through a portable yoke system. Black iron particles were sprayed with liquid suspension, enabling the free flow of particles over the workpiece surface. Fine iron particles were accumulated at the poles of a magnet. However, in any discontinuity/crack, iron particles accumulated at the flaw/crack location and followed the same orientation as a flaw/crack pattern, making visible the cracks highlighted in Figure 8. This indicates that cracks are randomly distributed, as can be seen by the naked eye. MT verified the results of UT by highlighting subsurface cracks. However, the cause of cracks and the nature of foreign particles could not be detected through magnetic particle testing. So, further investigation was required to find the root causes of sub-surface cracks or non-metallic inclusions.

#### 3.2.3. Microscopic Analysis and Testing

A careful and precise surface was prepared to detect the internal structure and entrapment details of foreign inclusions. The entrapment of non-metallic inclusions and internal cracks was studied from the viewpoint of chemical composition, as well as exogenous particles inside the final structure after ingot casting of 42CrMo4. Therefore, the surface of the samples was scrutinized with a microscope and scanning electron microscopy (SEM). The microscope passes an electron beam through the specimen by exploring the internal microstructural features; contrasts in the image are produced by differences in beam scattering or diffraction produced between various elements of the microstructure or defect.

**Figure 9:**
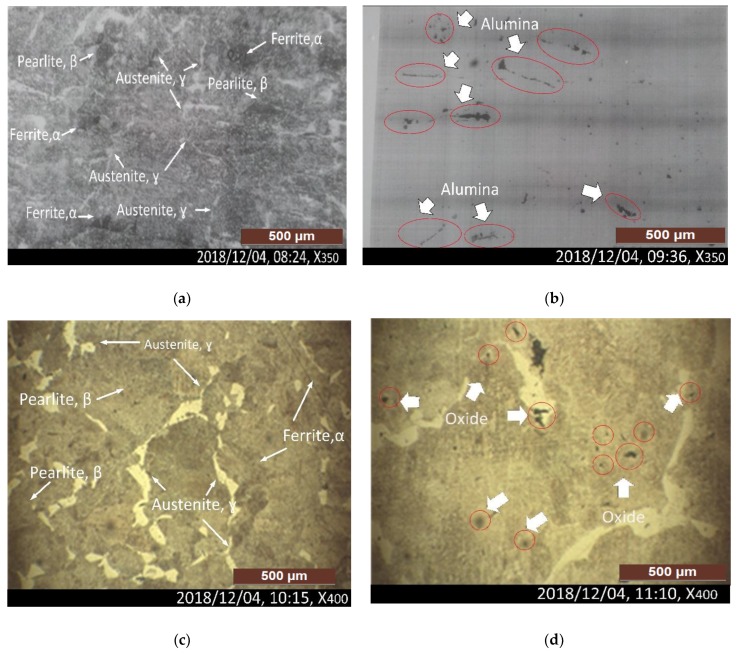
(**a**) As-cast structure of ferrite within the fine pearlite matrix and at prior austenite grain boundaries, at a magnification of ×350; (**b**) Not-etched micro alumina-type inclusions are observed as per ASTM E-45, at a magnification of x350; (**c**) A fine matrix structure of ferrite within the fine pearlite matrix and at prior austenite grain boundaries, at a magnification of x400; (**d**) Not-etched micro oxide-type inclusions, at a magnification of ×400.

Figure 9a depicts that the as-cast structure contains ferrite within a fine pearlite matrix and at prior austenite grain boundaries. The randomly distributed grain structure depicts that there is no issue in the grain size or chemical composition at a magnification of ×350. The grain size was determined in steel by ASTM (American Standard for Testing and Materials). Thus, steel with N = 6 has an average of 32 grains in an area of 1 sq. At ×100. On the surface of the 42CrMo4 sample, a significant exogenous inclusion of aluminium oxide (Al_2_O_3_) was identified (red circles) on the surface and subsurface, which agreed well with the ASTM E-45 standard presented in Figure 9b. Similarly, at a higher magnification (x400), Figure 9c underscores the ferrite within fine pearlite at prior austenite grain boundaries, depicting the random distribution of microstructure components and confirming the composition. Also, small exogenous oxide-type inclusions were identified by comparing the inclusion trends with ASTM E-45, presented in Figure 9d.

**Figure 10:**
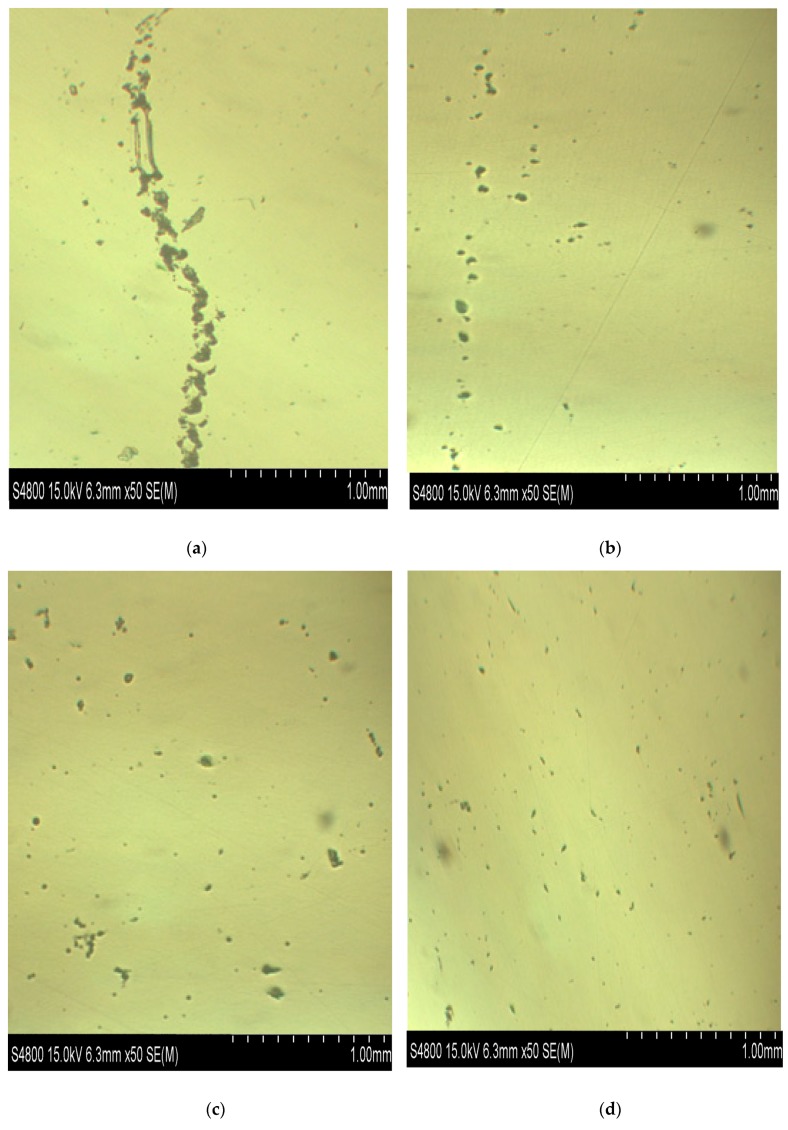
(**a**,**b**) Microstructure of sample indicating alumina-type inclusions, as per ASTM E-45, (**c**,**d**) Microstructure of sample indicating oxide-type inclusions, as per ASTM E-45.

After the identification of alumina and oxide-type inclusions through ASTM E-45, SEM images were attained for further verification of the inclusions. The SEM images depicted alumina and oxide-type inclusions as foreign particles. Figure 10a,b depicts the alumina foreign particle inclusions. Figure 10a depicts the heavy series of alumina-type inclusions, with a thickness of approximately 15μm. The depicted trend (Figure 10a) is compared with the Jernkontoret (JK) chart to determine the foreign particle contents in steel. The trend lies under alumina-type heavy series and level-3 reduction of standard E-45, by the permission of ASTM (American Standard of Testing and Materials). Figure 10b underscored the alumina-type inclusions with thin series having a thickness of approximately 9μm. The specific trend lies under alumina-type thin series and level-3 reduction, according to standard E-45.

Similarly, Figure 10c, d depicts the oxide-type inclusions. Figure 10c depicts the oxide-type inclusions under heavy series with a thickness of approximately 12μm. The specific trend of globular type oxide is compared with the Jernkontoret (JK) chart to verify the nature of the inclusions in the steel. The randomly distributed globular oxides showed no specific trend under oxide-type heavy series level-3, as per standard E-45 by the permission of ASTM. Besides, Figure 10d highlights oxide-type inclusions under thin series with a thickness of approximately 8μm. This is randomly distributed globular type oxide level-3, according to standard E-45. The provided microstructure images for these samples offered some information to judge the internal structure and induced surface characteristics. However, in order to be more compressive more details are still required, and thus macroscopic and macro-etching analyses are presented in the following section.

#### 3.2.4. Macroscopic Study/Macro-Etching Analysis

**Figure 11:**
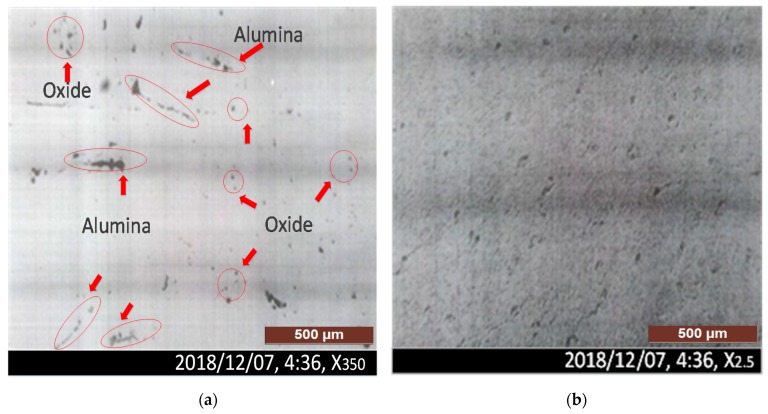
(**a**) Micro-inclusions, oxide and alumina-type inclusions, observed as per ASTM E-45 (**b**) The specimen was hotly etched in HCl (1:1), as inclusions were observed and randomly distributed as per ASTM E-381. The random condition is R-5.

Figure 11a depicts the specimen under macroscopic observation, which revealed oxide and alumina-type inclusions, observed as per ASTM E-45 [37]. The specimen was polished without etching to observe the non-metallic inclusions. Findings depicted the presence of randomly distributed alumina and oxide-type inclusions. Besides, the sample was hotly etched in HCl (1:1), as inclusions were observed, randomly distributed as per ASTM E-381 [38]. The random condition is presented by R-5 (Figure 11b). By comparing the metallographic tests with ASTM standards, oxide and alumina-type inclusions were recognized. The refractory material of the ladle and roof of the furnace were the sources from which alumina-type inclusions were transferred to casting material. Low-quality flux was the primary source of oxide-type inclusions. These non-metallic inclusions have a significant contribution to shaft rejection [39]. By applying standardized procedures at each step, the failures can be reduced to a minimum.

Microscopic testing, non-etched and hot-etched, was carried out to possibly detect the reasons associated with the rejection of the sugar mill shaft. The alumina and oxide-type foreign particles and microcracks, which were randomly distributed, indicated possible reasons for inclusions in the rough machined sugar mill shaft. Among numerous possible reasons for rejection, the results concluded that non-metallic inclusions, such as alumina and oxide-type inclusions, added through furnace hot-top and refractory material of the lining in the furnace, were the critical reasons that led to the shaft rejection.

## 4. Root Cause Analysis and Implementation

The widely used optimization techniques are lean manufacturing tools, total productive maintenance, and 6σ. These include failure mode effects analysis, root cause analysis (RCA), Ishikawa diagrams, and Pareto charts [40]. RCA has already been in place within various fields for over 60 years. The present approach is based on the lean production concept, i.e., “do more with fewer resources” and “do it better.” It serves as an essential tool to identify the original, most authentic cause or causes of an extremely complex problem. Based on this analysis, the organization can diminish the problem or avoid similar errors in the future [41].

The past trend of shaft rejection was collected to find the possible causes of rejections. The statistical data of the last 32 sugar mill shafts manufactured in heavy industry were collected to identify the significant causes of rejection in the past. A comprehensive data collection depicted that 50% shafts were rejected owing to the presence of non-metallic inclusions, 31.3% due to piping, and the remaining 18.7% due to surface cracks (Table 4).

**Figure 12:**
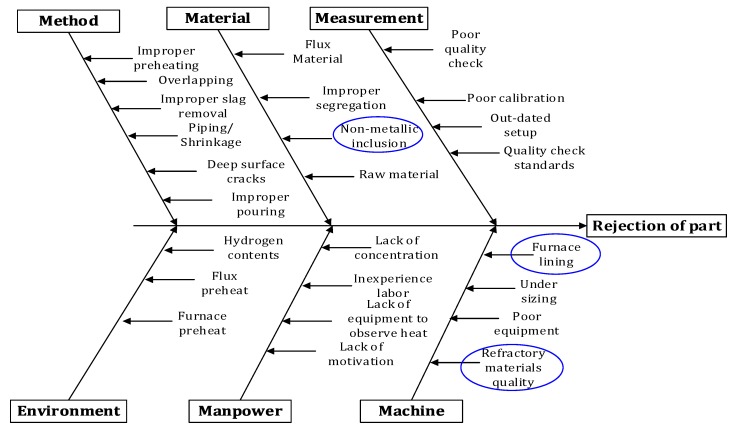
Cause and effect diagram highlighting the causes of shaft rejection.

Jayswal et al. [42] used a Fishbone diagram, which is a robust tool for detecting the possible causes of defects. Therefore, a similar methodology was implemented in this research. It helped to reach the root cause of the problem, i.e., the rejected mill drive shaft. By using a Fishbone diagram, it is possible to identify the main possible causes, which are associated with hydrogen contents, improper segregation of raw material, non-metallic inclusions, piping, lack of concentration of employees, furnace lining, inexperienced employees, improper pouring, deep surface cracks and so on. This methodology was validated for the mill drives shaft 42CrMo4, having the length of 2695 mm and diameter of 635 mm. The results permit the indication of the root causes of defects in order to avoid product rejection during the quality tests. The primary purpose of RCA is to find the underlying source of the observed symptoms of a problem [43]. The RCA technique was used in this research to identify and eliminate the possible causes of defects. The main purpose of RCA in this research work was to determine the key factors which affect the working environment and root causes of failures. This can be achieved by observing the history of previous events. In this way, the production efficiency can be improved by eliminating the major causes of failures. Root causes of failures and deviations from qualification standards in manufacturing are usually identified based on existing on-site expert knowledge regarding causal relationships between process steps and the nature of failures and deviations [44]. Identification and backtracking of root causes for said failures and deviations is exceptionally beneficial in order to avoid recurrence of such failures in the future. Tolerance analysis is an important parameter to determine the performance of the manufacturing process. It enables the measurement of the maximum possible variation of a quality feature in a production process [45]. The quality of the workpiece is paramount, and sometimes it becomes the primary constraint for productivity. The quality of the parts depends upon many direct and indirect factors (Figure 12), and those with poor quality are rejected during the production. Figure 12 highlights the factors such as methods, materials, measurement, environment, and other evaluation elements that affect the quality of a workpiece, such as the one tested in this study. Based on microstructure and SEM analysis, the root causes of rejection were highlighted. The proposed approach in this work is mainly based on achieving an integrated and solid analysis of such evaluation elements, and this approach showed a promising role in highlighting all possible problems/challenges associated with each element. Therefore, the single and interactional effects related to these problems were obtained, and this can support the decision to accept or reject any part by applying such a proper and practical approach. It should be stated that the proper application of such a technique and methodology helped to prevent such failure problems, and it is found that the reduction in the rejection rate for defective production was 7.6% after implementing the proposed failure detection approach.

## 5. Conclusions

In this study, the main causes of the rejection of a sugar mill roller shaft after rough machining were reported. Non-destructive and destructive tests were performed to identify the reasons for potential rejection. Non-destructive tests implemented here were X-ray spectroscopy, ultrasonic and magnetic particle testing, while destructive testing involved micro and macro crystallographic tests. The non-destructive evaluation demonstrated the formation of subsurface cracks and non-metallic inclusions at a specific depth and length in the rough machined ingot. While destructive testing provided the nature of non-metallic inclusions, the crack trends were acquired from the HCl-etched samples at different magnifications. The crack patterns were validated against industrial standards; some further Al2O3 and oxide-type inclusions were identified as well. The cause/effect diagram highlighted the possible origin of inclusions, which are the flux material, furnace lining, quality of refractory material, pre-heating of raw material, and so on. Based on the highlighted possible causes, the furnace lining and quality of refractory material were considered as the most critical parameters that led to the rejection of the final part. Nevertheless, the inclusions in the casting process are considered typical material defects. The implemented approach in this work permits the achievement of an integrated and reliable analysis. It is a very promising tool because it permits the highlighting of all the possible problems/challenges associated with each element.

Furthermore, as a correction process, the furnace lining, lining of the hot-top of the furnace and ladle were replaced with a better quality of refractory material. The feedback was collected after two months, and the rejection rate was reduced to 6.67% as only one shaft was rejected out of 15 during the process of the current research. Through a proper implementation that makes a quality standard for the entire system procedures, the rejection rate can be reduced further. These recommendations were implemented in heavy mechanical industry [46]. The drastic change in the rate of rejection was associated with the identification of root causes of failure and moving the system towards the application of standardized procedures. Further, by controlling the percentage of inclusions/impurities at each step, the rejection rate can be reduced as well. Moreover, waste management also plays an important role in manufacturing processes. To sum up, the single and interactional effects were obtained, which support the decision to accept or reject any part by applying such a proper and practical approach. It should be stated that the proper application of such a technique and methodology helped to prevent some failure problems. It is found that the reduction in the rejection rate for defective production was up to 7.6% after implementing the proposed failure detection approach.

The following recommendations are provided to heavy mechanical industry based on root cause analysis:

- Raw material should be properly segregated.
- The scrap should be shredded to break raw material into small pieces.
- The temperature of the furnace should be attained as nearly equal to the hot ingot temperature.
- There should be uniform pre-heating of the shaft before forging to avoid thermal stresses.
- The furnace lining should be maintained to avoid heat losses.
- Anti-hydrogen treatment can be avoided by introducing a vacuum degassing facility.
- A pyrometer should be used to measure high temperature.
- Qualification checks should be incorporated at each step.
- Equipment should be maintained properly to reduce cycle time.
- Top management should ensure the satisfaction level of their employees.

## Figures and Tables

**Table 1:** The standard chemical composition of 42CrMo4.

Chemical Properties	Name of the Components						
	C%	Mn%	Si%	S%	P%	Mo%	Cr%
	0.38–0.45	0.6–0.90	0.15–0.40	Max 0.035	Max 0.025	0.15–0.3	0.9–1.20
**Mechanical properties**	Tensile strength		980 N/mm^2^	Yield Strength	850 N/mm^2^	Material Elongation	14% (Min)

**Table 2:** The dimensions of the step-type flat bottom hole during the plotting of a distant amplitude correction (DAC) curve.

Step Height (mm)	Surface Distance of Flat Bottom Hole (FBH) (mm)	FBH Diameter (mm)	Depth of FBH (mm)
150	125	6	25
275	250		
400	375		

**Table 3:** Chemical composition of the mill drive shaft.

Chemical Composition	Name of the Components						
	C%	Mn%	Si%	S%	P%	Mo%	Cr%
Sample-1	0.38	0.82	0.349	0.028	0.02	0.144	1.0
Sample-2	0.371	0.81	0.357	0.030	0.018	0.152	0.98
Sample-3	0.384	0.80	0.341	0.033	0.025	0.145	0.97
Sample-4	0.367	0.84	0.336	0.035	0.021	0.156	1.02
Mechanical Properties	Tensile strength		965 N/mm^2^	Yield Strength	815 N/mm^2^	Elongation	12% (Min)

**Table 4:** Past data of shaft rejection based on ultrasonic testing**.**

Serial. No	Nature of Defects	Percentage
1	Non-metallic inclusions and subsurface cracks	50%
2	Piping	31.3%
3	Surface cracks	18.7%

## References

[1] Rathod C.T., Rathod W.S. (2012). Design, and analysis of two roller sugar mill using fea techniques. Int. J. Sci. Eng. Technol..

[2] Marín J.J. (2005). Fracture Mechanics Approach of Repaired Top Roll Shafts in Sugar Cane Mills. J. Mech. Behav. Mater..

[3] Shinde V.V., Swami M.C., Parmeshwar P. (2015). Weight Reduction and Analysis of Sugar Mill Roller Using FEA Techniques. Int. J. Latest. Trends Eng. Tech..

[4] Rivas J.S., Coronado J.J., Gómez A.L. (2006). Tribological aspects for the shafts and bearings of sugar cane mills. Wear.

[5] ELSawy E.E., EL-Hebeary M.R., El Mahallawi I.S. (2017). Effect of manganese, silicon and chromium additions on microstructure and wear characteristics of grey cast iron for sugar industries applications. Wear.

[6] Kakade A.B., Ingole S.P., Bhatt D.V., Menghani J.V. (2013). Tribological behavior of sugar mill roller shaft in laboratory simulated conditions. Wear.

[7] Kakade A., Ingole S., Bhatt D.V., Menghani J.V. (2017). Tribochemistry of Sugar Mill Roller Shaft Materials. Mater. Today Proc..

[8] Coombs J. (2014). Handbook of Cane Sugar Engineering.

[9] Sarkar B., Cárdenas-Barrón L.E., Sarkar M., Singgih M.L. (2014). An economic production quantity model with random defective rate, rework process and backorders for a single stage production system. J. Manuf. Syst..

[10] Jahanshahi A.A., Gashti M.A., Mirdamadi S.A., Nawaser K., Khaksar S.M. (2011). Study the effects of customer service and product quality on customer satisfaction and loyalty. Int. J. Hum. Soc. Sci..

[11] Dudek-Burlikowska M., Szewieczek D. (2009). The Poka-Yoke method as an improving quality tool of operations in the process. J. Achiev. Mater. Manuf. Eng..

[12] Goetsch D.L., Davis S.B. (2014). Quality Management for Organizational Excellence.

[13] Agunsoye J.O., Isaac T.S., Awe O.I., Onwuegbuzie A.T. (2013). Effect of silicon additions on the wear properties of grey cast iron. J. Miner. Mater. Character. Eng..

[14] Bowler J.R., Bowler N. (2002). Evaluation of the magnetic field near a crack with application to magnetic particle inspection. J. Phys. D Appl. Phys..

[15] Rokhlin S.I., Kim J.Y., Xie B., Zoofan B. (2007). Nondestructive sizing and localization of internal microcracks in fatigue samples. NDT E Int..

[16] Kukla D., Bałkowiec A., Grzywna P. (2014). Evaluation of microstructural changes of S235 steel after rolling on the basis of microscopic observations and eddy current non-destructive method. Adv. Mater. Sci..

[17] Zhang L., Thomas B.G. (2006). State of the art in the control of inclusions during steel ingot casting. Metall. Mater. Trans. B.

[18] Fuller R.W., Ehrgott J.Q., Heard W.F., Robert S.D., Stinson R.D., Solanki K., Horstemeyer M.F. (2008). Failure analysis of AISI 304 stainless steel shaft. Eng. Fail. Anal..

[19] Zhao L.H., Xing Q.K., Wang J.Y., Li S.L., Zheng S.L. (2019). Failure and root cause analysis of vehicle drive shaft. Eng. Fail. Anal..

[20] Ni T.W., Ding Q., Yang Z.G., Zheng H.L., Lou X. (2018). Failure analysis on premature fracture of boric acid recycle pump shaft in 1000 MW nuclear power plant. Eng. Fail. Anal..

[21] Zerbst U., Madia M., Klinger C., Bettge D., Murakami Y. (2019). Defects as a root cause of fatigue failure of metallic components. II: Non-metallic inclusions. Eng. Fail. Anal..

[22] Federici M., Straffelini G., Gialanella S. (2017). Pin-on-disc testing of low-metallic friction material sliding against HVOF coated cast iron: Modelling of the contact temperature evolution. Tribol. Lett..

[23] Pavan A.H., Vikrant K.S., Swamy M., Jayaraman G. (2013). Root cause analysis of bowl-mill pinion shaft failures. Case Stud. Eng. Fail. Anal..

[24] Prasanthi T.N., Sudha C., Parameswaran P., Punniyamoorthy R., Chandramouli S., Saroja S., Rajan K.K., Vijayalakshmi M. (2013). Failure analysis of a 304 steel component aged at 623 K. Eng. Fail. Anal..

[25] Mapelli C. (2008). Non-metallic inclusions and clean steel. Met. Ital..

[26] Saberifar S., Mashreghi A.R., Mosalaeepur M., Ghasemi S.S. (2012). The interaction between non-metallic inclusions and surface roughness in fatigue failure and their influence on fatigue strength. Mater. Des..

[27] Yang Z.G., Yao G., Li G.Y., Li S.X., Chu Z.M., Hui W.J., Dong H., Weng Y.Q. (2004). The effect of inclusions on the fatigue behavior of fine-grained high strength 42CrMoVNb steel. Int. J. Fatigue.

[28] Khan A.M., Jamil M., Mozammel M., Yurievich P.D., Rashitovich G.V., Munish K.G., He N. (2018). Multi-objective optimization for grinding of AISI D2 steel with Al2O3 wheel under MQL. Materials.

[29] Chan K.S. (2003). A microstructure-based fatigue-crack-initiation model. Metall. Mater. Trans. A.

[30] Chan K.S. (2010). Roles of microstructure in fatigue crack initiation. Int. J. Fatigue.

[31] Wang Q.Y., Bathias C., Kawagoishi N., Chen Q. (2002). Effect of inclusion on subsurface crack initiation and gigacycle fatigue strength. Int. J. Fatigue.

[32] Hong Y., Zhao A., Qian G., Zhou C. (2012). Fatigue strength and crack initiation mechanism of very-high-cycle fatigue for low alloy steels. Metall. Mater. Trans. A.

[33] Santosh Y.S. (2015). Static Structural Analysis of Conventional Sugar Mill Roller Shaft for Ø40”x 80” Milling Plant. Int. J. Late. Trend. Eng. Tech..

[34] Kalinichenko N.P., Kalinichenko A.N., Lobanova I.S., Zaitseva A.A. (2016). Universal reference test blocks for liquid penetrant testing. Eng. Mater.

[35] Glazkov Y.A. (2004). Peculiarities of the certification of reference specimens for liquid-penetrant testing. Russ. J. Nondestr. Test.

[36] Adams R.D., Cawley P.D. (1988). A review of defect types and nondestructive testing techniques for composites and bonded joints. NDT Int..

[37] ASTM International (2018). ASTM E45-18a Standard Test Methods for Determining the Inclusion Content of Steel.

[38] ASTM International (2017). ASTM E381-17 Standard Method of Macroetch Testing Steel Bars, Billets, Blooms, and Forgings.

[39] Zeng Y.P., Xu C.M., Dong J.X., Zhang M.C., Xie X.S. (2005). Micro behavior study of non-metallic inclusion in high strength shaft steel. Eng. Mater..

[40] Card D.N. Myths and strategies of defect causal analysis. Proceedings of the Pacific Northwest Software Quality Conference.

[41] Sobel K. (2017). Root Cause Analysis: Parsing Complex Challenges in Academic Libraries. J. Academ. Librar..

[42] Jayswal A., Li X., Zanwar A., Lou H.H., Huang Y. (2011). A sustainability root cause analysis methodology and its application. Comput. Chem. Eng..

[43] Schoenfisch J., Meilicke C., von Stülpnagel J., Ortmann J., Stuckenschmidt H. (2018). Root cause analysis in IT infrastructures using ontologies and abduction in Markov Logic Networks. Inf. Syst..

[44] Lokrantz A., Gustavsson E., Jirstrand M. (2018). Root cause analysis of failures and quality deviations in manufacturing using machine learning. Procedia CIRP.

[45] Mahshid R., Mansourvar Z., Hansen H.N. (2018). Tolerance analysis in manufacturing using process capability ratio with measurement uncertainty. Precis. Eng..

[46] Hmc Heavy Mechanical Complex. http://hmc.com.pk/.

